# Dynamics of the quorum sensing switch: stochastic and non-stationary effects

**DOI:** 10.1186/1752-0509-7-6

**Published:** 2013-01-16

**Authors:** Marc Weber, Javier Buceta

**Affiliations:** 1 Computer Simulation and Modelling (Co.S.Mo.) Lab, Parc Científic de Barcelona, C/ Baldiri Reixac 4, 08028 Barcelona, Spain

**Keywords:** Quorum sensing, Noise, Stochastic modeling, Vibrio fischeri, Autoinducer, Synchronization

## Abstract

**Background:**

A wide range of bacteria species are known to communicate through the so called quorum sensing (QS) mechanism by means of which they produce a small molecule that can freely diffuse in the environment and in the cells. Upon reaching a threshold concentration, the signalling molecule activates the QS-controlled genes that promote phenotypic changes. This mechanism, for its simplicity, has become the model system for studying the emergence of a global response in prokaryotic cells. Yet, how cells precisely measure the signal concentration and act coordinately, despite the presence of fluctuations that unavoidably affects cell regulation and signalling, remains unclear.

**Results:**

We propose a model for the QS signalling mechanism in *Vibrio fischeri* based on the synthetic strains *lux01* and *lux02*. Our approach takes into account the key regulatory interactions between LuxR and LuxI, the autoinducer transport, the cellular growth and the division dynamics. By using both deterministic and stochastic models, we analyze the response and dynamics at the single-cell level and compare them to the global response at the population level. Our results show how fluctuations interfere with the synchronization of the cell activation and lead to a bimodal phenotypic distribution. In this context, we introduce the concept of precision in order to characterize the reliability of the QS communication process in the colony. We show that increasing the noise in the expression of LuxR helps cells to get activated at lower autoinducer concentrations but, at the same time, slows down the global response. The precision of the QS switch under non-stationary conditions decreases with noise, while at steady-state it is independent of the noise value.

**Conclusions:**

Our *in silico* experiments show that the response of the LuxR/LuxI system depends on the interplay between non-stationary and stochastic effects and that the burst size of the transcription/translation noise at the level of LuxR controls the phenotypic variability of the population. These results, together with recent experimental evidences on LuxR regulation in wild-type species, suggest that bacteria have evolved mechanisms to regulate the intensity of those fluctuations.

## Background

Bacteria, long thought having a solitary existence, were found to communicate with one another by sending and receiving chemical messages [1]. Their communication mechanism results in the ability to synchronize the activity of the colony as a whole. The latter leads to a coordinated behaviour that in some cases resembles that of multicellular organisms, e.g. the so-called community effect during development [2]. Thus, by means of the quorum sensing (QS) mechanism, cells produce, export, and import signalling molecules (autoinducer). As the colony grows, more cells produce and export autoinducer, leading to an increasing concentration of the signalling molecule in the environment and in the cells. Upon reaching a concentration threshold, the autoinducer activates the expression of QS-controlled genes therefore coordinating the cells in a density-dependent manner. Importantly, QS controls a number of relevant phenotypic changes in bacteria as for example the virulence in *S. aureus*[3]. In addition, it has become a model system for studying the emergence of coordinated behaviour in communicating cells. All in all, QS has opened a research field with promising technological applications [4], as for example, the environmentally controlled invasion of cancer cells [5].

The QS systems in gram-negative bacteria share a core network architecture. In this regard, a characteristic model system is the LuxR/LuxI regulatory network in *Vibrio fischeri*[6]. LuxR protein is an autoinducer-dependent activator of the lux operon that drives the autocatalytic expression of *luxR* and of the autoinducer synthase, *luxI*, together with that of the genes responsible for the production of bioluminescence. The up-regulation of *luxI* increases the production of autoinducer molecules that in turn activates further gene expression. The resulting positive feedback loop leads to a bistable switch-like behaviour depending on the concentration of the autoinducer as shown by *in silico*[7-9] and *in vivo* experiments [10,11]. Such switch-like behaviour has been observed at the population level by measuring the average gene expression level. However, how individual cells behave remains puzzling. In fact, as observed in *Vibrio harveyi*[12], *Vibrio fischeri*[13], *Pseudomonas aeruginosa*[14], and *luxI*/*luxR-GFP* strains of *E. coli*[15], the cellular response to QS signals seems to be highly heterogeneous at the level of the distribution of both the population phenotype and the response times of individual cells.

A number of studies have shown that noise plays an important role in bistable systems [16-18]. Therefore, the aforementioned heterogeneity may be caused by the random fluctuations that unavoidably affect cell regulation and signalling. This poses the intriguing question of how cells achieve a coordinated response in the presence of noise. Indeed, the QS mechanism may produce a robust and synchronized behaviour at the level of the population both experimentally [19] and theoretically [20]. However, how this behaviour at the collective level arises from the stochastic dynamics of individual cells is still an open question. At the end, in the framework of QS, a collective response means a precise information exchange in the colony. Consequently, how can a bacterial population estimate its number of constituents precisely if such information is fuzzy at the single cell level? Herein, we shed light on this problem and investigate how noise affects the QS transition both at the level of individual cells and at the level of the cell population.

In the context of QS modelling, most research has focused on the understanding of the intracellular circuit [7-11,21-24], i.e. single cell studies, while few of them have considered an ensemble of communicating cells [25-28]. Yet, so far no study has taken into account the coupling of the signalling mechanism at the single cell and collective levels by stochastic means together with realistic dynamics of the proliferation process. In this work, we model the QS mechanism by using both deterministic and stochastic approaches and taking into account the key regulatory interactions between LuxR and LuxI, the autoinducer transport, the cellular growth and the division dynamics. Our results indicate that the cell response is highly heterogeneous and that noise in the gene expression of *luxR* is the main factor that determines this variability. Moreover, we show that the transition of the QS switch near the critical concentration of autoinducer is very slow compared to other characteristic temporal scales of the process and that, as a consequence, the non-stationary effects are crucial for setting a precise switch. As we show further below, the dilution due to cell growth and division is a key element required for an in-depth understanding of the QS response dynamics. In addition, we demonstrate that noise, depending on the cell density, can either prevent or promote phenotypic changes indicating a beneficial role played by stochasticity. Altogether, we find that the precision of the QS switch for determining the number of cells in the colony is highly dynamic and context dependent, which in turn favors adaptability.

## Methods

### Modelling of the LuxI/LuxR gene regulatory network

**Figure 1:**
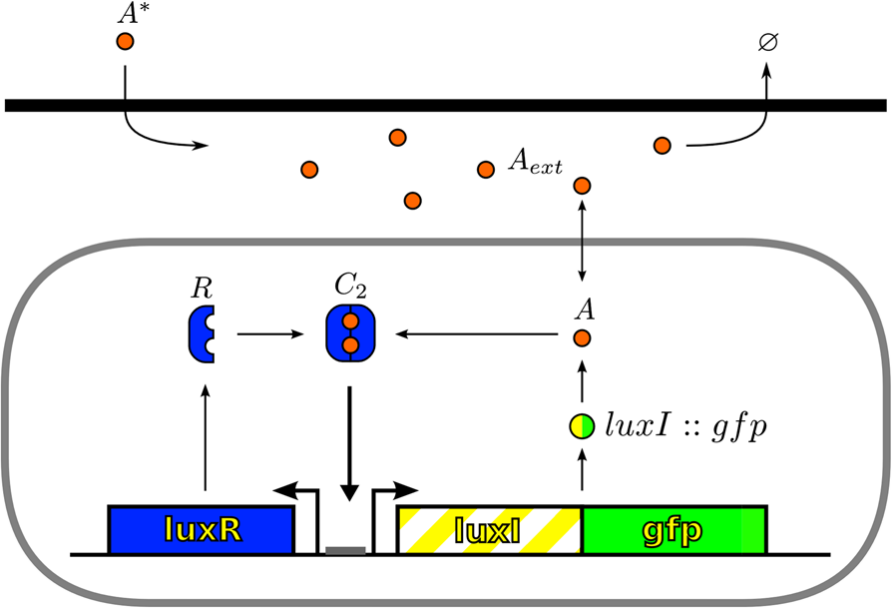
**Scheme of the LuxI/LuxR regulatory network.** The LuxR (*R*) protein activates the operon upon binding to autoinducer molecules (*A*). The *lux01* operon lacks the *luxI* gene and therefore cells cannot produce their own autoinducer and exogenous signalling molecules are needed to activate the expression of *luxR* and GFP [10]. On the other hand, the *lux02* operon carries a *luxI::gfp* fusion and allows for the production of autoinducer and self-induction (see text for details).

The regulatory interactions that control the wild-type lux operon are more complex than first thought [29]. Those include both positive and negative regulation of the *luxR* gene depending on the concentration of the autoinducer [30]. Simplified synthetic constructs, such as *lux01* and *lux02*[10], retain the minimal *luxI/luxR* regulatory motif and lack the structural genes responsible for light emission that may also play a regulatory role, e.g. *luxD*[31]. Still, these constructs reproduce the main features of the wild-type operon as revealed by GFP tags reporting the promoter activity [10]. In addition, *lux01* and *lux02* constructs allow to perform controlled experiments that have shed light on the wild-type dynamics and its regulatory interactions. Herein, we follow this approach and focus on the *lux01* and *lux02* constructs as well characterized examples of the behaviour of the wild-type operon. The *lux01* operon lacks the *luxI* gene and only *gfp* is transcribed in that direction. On the other hand, the *lux02* operon carries a *luxI::gfp* fusion. Accordingly, *lux01* cells cannot produce their own autoinducer and the induction in that case is driven by adding exogenous autoinducer to the medium. Figure 1 shows schematically the regulatory interactions we consider in our model. The autoinducer molecules (*A*) are produced due to the action of their synthetase, LuxI, and bind to the cytoplasmic protein LuxR (*R*) creating a complex (*C*_2_). The latter binds to the promoter region activating both the transcription of *luxI::gfp* (only *gfp* in the case of *lux01*) and *luxR*. Signalling molecules can diffuse passively in and out the cell and contribute to increase the external concentration of the autoinducer (*A*_*ext*_) that can be eventually modified by an external influx of molecules (*A*^∗^) and a dilution protocol (see below). In our model we consider that signalling molecules degrade at the same rate whether they are cytoplasmic or not. Finally, we consider a *DNA* duplication process. Such modelling scheme can be formally written as a set of chemical reactions:

$$\begin{array}{rcll} \text{DNA} & \overset{\alpha_{R}k_{R}}{\to } & \text{DNA}+\text{mRN}A_{\text{luxR}} & \\ \text{DNA} & \overset{\alpha_{I}k_{I}}{\to } & \text{DNA}+\text{mRN}A_{\text{luxI}::\text{gfp}} & \\ \text{DNA}\cdot {\left(\text{luxR}\cdot A\right)}_{2} & \overset{k_{R}}{\to } & \text{DNA}\cdot {\left(\text{luxR}\cdot A\right)}_{2}+\text{mRN}A_{\text{luxR}} & \\ \text{DNA}\cdot {\left(\text{luxR}\cdot A\right)}_{2} & \overset{k_{I}}{\to } & \text{DNA}\cdot {\left(\text{luxR}\cdot A\right)}_{2}+\text{mRN}A_{\text{luxI}::\text{gfp}} & \\ \text{mRN}A_{\text{luxR}} & \overset{p_{R}}{\to } & \text{mRN}A_{\text{luxR}}+\text{luxR} & \\ \text{mRN}A_{\text{luxI}::\text{gfp}} & \overset{p_{I}}{\to } & \text{mRN}A_{\text{luxI}::\text{gfp}}+\text{luxI}::\text{gfp} & \\ \text{luxI}::\text{gfp} & \overset{k_{A}}{\to } & A+\text{luxI}::\text{gfp} & \\ \text{luxR}+A & \overset{k_{1}^{-}/K_{d1}}{\underset{k_{1}^{-}}{↔}} & \text{luxR}\cdot A & \\ 2\left(\text{luxR}\cdot A\right) & \overset{k_{2}^{-}/K_{d2}}{\underset{k_{2}^{-}}{↔}} & {\left(\text{luxR}\cdot A\right)}_{2} & \\ {\left(\text{luxR}\cdot A\right)}_{2}+\text{DNA} & \overset{k_{\text{lux}}^{-}/K_{\text{dlux}}}{\underset{k_{\text{lux}}^{-}}{↔}} & \text{DNA}\cdot {\left(\text{luxR}\cdot A\right)}_{2} & \\ A & \overset{D}{\underset{\text{rD}}{↔}} & A_{\text{ext}} & \\ A & \overset{d_{A}}{\to } & \emptyset & \\ A_{\text{ext}} & \overset{d_{A}}{\to } & \emptyset & \\ \text{mRN}A_{\text{luxR}} & \overset{d_{\text{mR}}}{\to } & \emptyset & \end{array}$$

(1) $$\begin{array}{rcll} \text{mRN}A_{\text{luxI}::\text{gfp}} & \overset{d_{\text{mI}}}{\to } & \emptyset & \\ \text{luxR} & \overset{d_{R}}{\to } & \emptyset & \\ \text{luxI}::\text{gfp} & \overset{d_{I}}{\to } & \emptyset & \\ {\left(\text{luxR}\cdot A\right)}_{2} & \overset{d_{C_{2}}}{\to } & \emptyset & \\ \text{luxR}\cdot A & \overset{d_{C}}{\to } & \emptyset & \\ \text{DNA} & \overset{\text{ln}(2)/\tau }{\to } & \text{DNA}+\text{DNA} & \\ \text{DNA}\cdot {\left(\text{luxR}\cdot A\right)}_{2} & \overset{\text{ln}(2)/\tau }{\to } & \text{DNA}\cdot {\left(\text{luxR}\cdot A\right)}_{2}+\mathrm{DNA.} & \end{array}$$

As revealed by the set of reactions (1), we assume that the regulatory complex (*luxR*·*A*)_2_ activates the transcription of *luxI* and *luxR* in opposite directions upon binding to the *DNA*. These reactions account for the main regulatory interactions of both *lux01* and *lux02* constructs. Since *lux01* lacks the *luxI* gene the autoinducer, *A*, cannot be synthesized, i.e. *k*_*A*_=0, and an exogenous supply of the signalling molecule is required to induce the system. The expression rates of *luxI* and *luxR* depend on the initiation rate of transcription, the speed of elongation, the length of the transcript, and the rate of translation and postmodification into functional proteins. We take into account the differences due to these intermediate processes in an effective manner by using different transcription/translation rates for the *luxR* and *luxI::gfp* genes. Note that we assume that there are basal transcriptional rates, *α*_*R*_*k*_*R*_ and *α*_*I*_*k*_*I*_, even though the regulatory complex (*luxR*·*A*)_2_ is not bound to the promoter region of the *DNA*. Still, since *α*_*R*_,*α*_*I*_≪1 (see parameter values below), the maximum transcriptional rates take place when the activator complex is bound.

### Deterministic and stochastic approaches: cell growth and division

**Figure 2:**
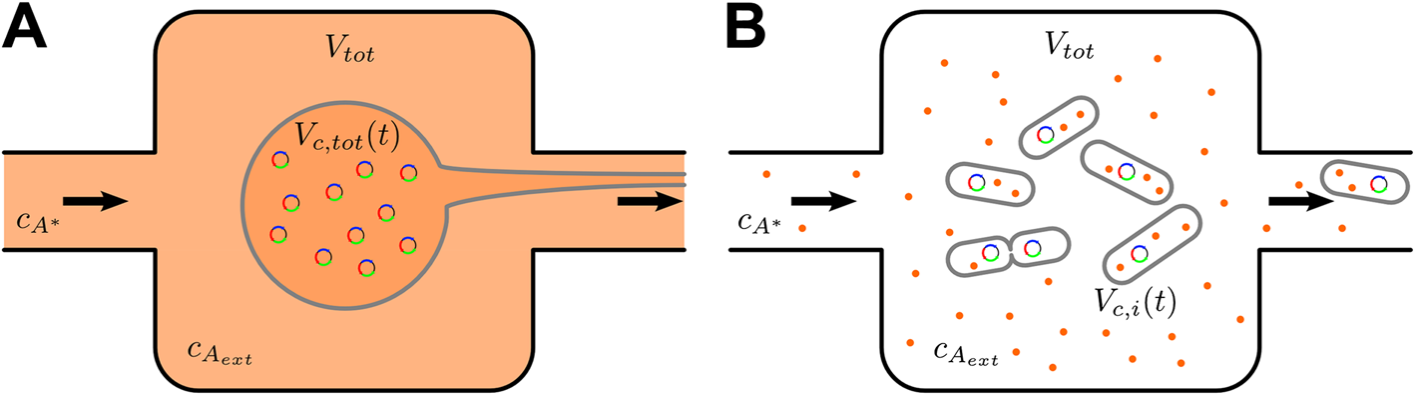
**Scheme of the deterministic and stochastic modelling approaches. A**: In the deterministic model, the population of cells is described by a unique volume with average and continuous concentrations of all species, including the DNA carrying the QS network (small circles). Cellular growth is also taken into account in this approach. **B**: In the stochastic model, cells are modelled as individual compartments that can grow and divide and all molecular species are represented as discrete entities. In both cases, **A** and **B**, we assume that all species are well-stirred inside the cells and in the medium. In order to maintain a constant cell density, as in the experiments we aim to model, we implement a dilution protocol. In the deterministic model the dilution removes continuously cytoplasmic material in order to compensate the cell growth. In the stochastic model individual cells are removed every time a new cell is born (see Additional file 2: Video S1).

The equations (1) lead to a Master equation description that can be sampled exactly by means of the Gillespie algorithm [32]. This approach is suitable for the characterization of the system at the single cell level. Complementary to this, if the number of molecules of the species is large enough such that the fluctuations can be neglected, a set of ordinary differential equations (ODEs) can be derived from Eqs. (1) (see Additional file 1: Text S1). The ODEs formalism is then appropriate to account for the behaviour at the colony level since noise averages out in that case. Herein we make use of both stochastic and deterministic descriptions as follows. As for the deterministic model, we consider that all cells share their cytoplasm in a *single* volume *V*_*c*,*tot*_(Figure 2). Chemical species *X* inside *the* cell are described by their concentration, *c*_*X*_, in *V*_*c*,*tot*_. Therefore, this model can only be used to study the dynamics of species averaged over all the cells in the population. From an experimental point of view, the population average can be measured determining the average bulk fluorescence of the *GFP* reporter of the cell culture by means of a fluorometer or by averaging the fluorescence data obtained with a flow cytometer.

We notice that our *in silico* experiments span up to 100 hours of cell culture growth in some cases (simulated experimental time, not computational time). Thus, regardless of the description, and in addition to the dynamics of the regulatory network, we also need to take into account the effects of cell growth. If cells are maintained in the exponential phase with doubling time *τ* then the dynamics of the volume of *the* cell is *V*_*c*,*tot*_(*t*)=*V*_0,*tot*_2^*t*/*τ*^. Where *V*_0,*tot*_=*NV*_0_, *N* being the number of cells in the colony and *V*_0_the volume of a single cell at the beginning of the cell cycle. As a consequence, the cellular growth introduces dilution terms, $-c_{X}\frac{\text{ln}(2)}{\tau }$, in the r.h.s. of the ODEs of all species, with the exception of the autoinducer in the medium *A*_*ext*_. On the other hand, cell division events lead to the duplication of the genetic material. The latter is taken into account by adding the term $+\frac{\text{ln}(2)}{\tau }\left(c_{\text{DNA}}+c_{\text{DNA}\cdot {\left(\text{luxR}\cdot A\right)}_{2}}\right)$ to the ODE that describes the concentration of *DNA*. This term compensates exactly for the cell growth dilution such that $c_{\text{DNA},\text{tot}}=c_{\text{DNA}}+c_{\text{DNA}\cdot {\left(\text{luxR}\cdot A\right)}_{2}}$, i.e. the total concentration of DNA, is kept constant.

In our simulations, as in the experiments we aim to reproduce, the cell density is kept constant. This can be achieved by means of an external dilution protocol (see below) that compensates for cell proliferation. We then keep the volume *V*_*c*,*tot*_ constant and define the external volume, *V*_*ext*_, such that the total volume of the cell culture reads *V*_*tot*_=*V*_*ext*_ + *V*_*c*,*tot*_. Accordingly, the parameter *r*, see equations (1), reads *r*=*V*_*c*,*tot*_/*V*_*ext*_. We assume that molecules are homogeneously distributed inside both the cytoplasm and the external volume (i.e. spatial effects are disregarded). Finally, the resulting ODEs are numerically integrated.

In order to study the role of noise in a population of cells communicating by QS, we build also a stochastic model of a population of bacteria. In this case, each bacterium is described as a single cell carrying a copy of the regulatory network. The ensemble of all the chemical reactions in all cells, including the diffusion reaction, are treated as one global system. We apply the Gillespie algorithm [32] to compute the time of the next reaction, choose the reaction channel from the list of all possible reactions and update the number of molecules according to the reaction stoichiometry. We model the system of cells as a global stochastic system in order to simulate as exactly as possible the stochastic dynamics of all chemical species, in particular that of autoinducer molecules. The noise in the signalling molecule originates from different sources: randomness in its synthesis by LuxI, fluctuations at the level of the number of molecules of LuxI, and randomness in the diffusion reaction of the autoinducer. The latter is particularly important since it leads to correlations between cells as follows. An autoinducer molecule can diffuse out of the cytoplasm of one cell into the medium, thereby increasing the number of molecules in the external volume by one; this increase in the level of *A*_*ext*_changes the probabilities of an autoinducer molecule to diffuse into any other cell. Thus, all the cells are coupled through the diffusion reaction. We note that while a possible optimization of the algorithm relies on parallelizing the code such that each cell evolves independently [25], this approximation is prone to introduce errors in the dynamics of the signalling molecule because the aforementioned correlations are neglected.

As mentioned above, cell growth introduces a dilution of the molecules in a cell. We implement cell growth in our stochastic model by allowing the volume of cell *i* to change in time as,

$$V_{c,i}(t)=V_{0}2^{t/\tau_{i}},$$

where *V*_0_is the volume of a cell at the beginning of the cell cycle (same for all cells), *τ*_*i*_is the duration of the cell cycle of cell *i*, and *t* is referred to the precedent division event. When *t*=*τ*_*i*_ the cell *i* has doubled its volume and a new division takes place. At this time the internal clocks and volumes of daughter cells are reset to zero and *V*_0_respectively. Moreover, when a cell divides, proteins, mRNAs and signalling molecules are binomially distributed [33] between daughter cells and one copy of the DNA is given to each cell. We note that regulatory complexes bound to the DNA are detached prior to the distribution between daughter cells. As in the case of the deterministic model, we assume that the cell density is maintained constant during experiments due to a compensational external efflux that wash away cells in the culture (see below). In relation to the effect of the cell volume of individual cells on the diffusion rate of the autoinducer, we note that in this case,

$$r_{i}\left(t\right)=\frac{V_{c,i}(t)}{V_{\text{tot}}-\overset{N}{\underset{j=1}{\sum }}V_{c,j}(t)}.$$

The duration of the cell cycle, *τ*_*i*_is different for each cell and is set independently after a division according to the following stochastic rule [34],

$$\tau_{i}=\lambda \tau +\left(1-\lambda \right)\tilde{\tau },$$

where *τ* and $\tilde{\tau }$ denote, respectively, the deterministic and stochastic components of the cell cycle duration, and *λ*∈[0,1] is a parameter that weights their relative importance. The stochastic component accounts for the period of time between events driven by a Poissonian process and satisfies an exponential distribution,

$$\rho \left(\tilde{\tau }\right)=\frac{e^{-\frac{\tilde{\tau }}{\tau }}}{\tau }.$$

In this way, we allow variability from cell to cell in regards of the duration of the cell cycle, yet setting a minimum cell cycle duration, *λτ*. According to these definitions, the average duration and standard deviation of the cell cycle are *τ* and (1−*λ*)*τ*respectively.

Finally, we notice that in principle the Gillespie algorithm needs to be adapted in order to take into account the time-dependent cell volume. The propensity of a second-order reaction at cell *i* at time *t* scales as *p*_*i*_(*t*)=*p*_0_*V*_0_/*V*_*c*,*i*_(*t*), where *p*_0_ stands for propensity of the reaction at division time when *V*_*c*,*i*_(0)=*V*_0_. The propensity *p*_0_are derived from the corresponding reaction rate, *k,* by dividing the latter by the initial cell volume, *p*_0_=*k*/*V*_0_. In addition to the change in the propensities of the reaction channels, the algorithm would also need to be adapted to compute the time till next reaction [35]. However, in our case, since all reactions rates are faster than the rate of variation of the cell volume, ∼1/*τ*, (see parameter values below) then the volume increase is negligible during the time interval until the next reaction takes place. Consequently, we can adiabatically eliminate the volume growth dynamics and safely assume that the volume-dependent propensities remain constant until the next reaction occurs. Summarizing, at a given time *t* we compute, as described above, the time-dependent propensities based on the volume of the cell at that time and, according to those, we determine the time at which the next reaction takes place, *t* + △*t*, following the standard Gillespie algorithm.

### Gene expression noise: burst size

During translation mRNA molecules are translated into proteins following a bursting dynamics [36-38]. The so-called burst size, *b*_*X*_, is defined as the ratio between the protein *X* production rate and the mRNA *X* degradation rate. It has been shown that *b*_*X*_ is directly related to the intensity of gene expression noise [36,39]. Thus, for the same average protein concentration, the larger *b*_*X*_ is, the more fluctuating expression dynamics is displayed by protein *X*. In our stochastic simulations we use the burst size *b*_*X*_as a parameter to tune the noise intensity at the level of *luxI* and *luxR* and study its effects. Unless explicitly indicated otherwise, the bursting size in the stochastic simulations is *b*_*R*_=*b*_*I*_=20.

### External dilution protocol

In controlled experimental setups it is advantageous to keep the cell density constant. This is carried out by means of an external dilution protocol that compensates for cell growth. Experimentally, this is usually achieved by periodic dilutions of the cell culture [10] or by a continuous flow of liquid medium in a chemostat or in a microfluidic device [40]. This procedure allows to measure the stationary concentration of the signalling molecule at a given cell density and/or to estimate the threshold of the QS collective response of a cell culture. Moreover, the external dilution is also important in order to maintain cells in the exponential growth phase and prevent depletion of nutrients in the medium. Additionally, the levels of the autoinducer can be controlled by adding/removing exogenous signalling molecules in/from the culture buffer. We implement those in our simulations as follows.

In the deterministic model, as shown in Figure 2, we assume a unique cell with volume *V*_*c*,*tot*_. Cell density is controlled by a continuous efflux that removes cytoplasm and culture medium at a rate that compensates exactly for the cell growth, such that the volume *V*_*c*,*tot*_remains constant. Concurrently, a continuous influx of equal and opposite rate brings fresh medium to the cell culture. In our *in silico* stochastic experiments, the efflux is reproduced by removing molecules, *A*_*ext*_, from the medium and washing away cells by “deleting” a cell picked at random in the population each time a new cell is born.

In our simulations, as in the experiments we aim to reproduce, the exogenous autoinducer concentration $c_{A}^{*}$ is the control parameter [10]. This means that the levels of autoinducer are controlled by varying the concentration of exogenous autoinducer in the dilution buffer (influx). The influx of exogenous autoinducer molecules, together with the efflux of culture medium, can be represented by the following reaction,

$$A_{\text{ext}}\overset{\gamma }{\underset{\gamma c_{A}^{*}V_{\text{tot}}}{↔}}\emptyset .$$

where *γ*=*ln*(2)/*τ*. That is, an efflux removes autoinducer molecules from the external volume at a rate *γ*and an influx introduces signalling molecules in the external volume at a rate $\gamma c_{A}^{*}V_{\text{tot}}$. In the deterministic description, the last equation leads to an additional term at the r.h.s. of the ODE for the concentration of *A*_*ext*_:$+\gamma \left(c_{A}^{*}\frac{V_{\text{tot}}}{V_{\text{ext}}}-c_{A_{\text{ext}}}\right)$. We notice that in our simulations, as in experiments, *V*_*tot*_/*V*_*ext*_≃1. In the absence of synthesis (e.g. *lux01*) and taking into account that the degradation is slower than the diffusion and the influx rate, it is easy to see that the concentration of autoinducer, both inside and outside *the* cell, tends to $c_{A}^{*}$: the desired control value of the autoinducer concentration (see Additional file 3: Figure S1).

### Parameters

The parameters used in our model are listed in Table 1. When possible, parameter values are fixed or estimated by using experimental measurements found in the literature. The rest of the parameters are fitted to the experimental data of [10] using the deterministic model to reproduce the main characteristics of the response curves of the *lux01* operon: a difference of two orders of magnitude in the level of expression of GFP between the low and the high states, a hysteresis effect in the range of autoinducer concentrations $0<c_{A}^{*}<15\;\text{nM}$, and a time to reach steady-state at full induction ($c_{A}^{*}=100\;\text{nM}$) shorter than 6 hours. In regards of the cell density, based on an estimate of the CFU/mL for an average OD of 0.5 for *E. coli* cells, we take a typical value of *c*_*N*_=5·10^8^cells/mL. Moreover, in order to keep the computational time within reasonable limits, we choose a system size of *N*=100 cells. After fixing the number of cells and the cell density, the total and external volumes are then respectively derived from the relations *c*_*N*_=*N*/*V*_*tot*_ and *V*_*ext*_=*V*_*tot*_−*NV*_0_, where *V*_*tot*_=2·10^−4^*μL*. Finally, for the case of the *lux02* operon there is one additional parameter that needs to be calibrated: the synthesis rate of the autoinducer, *k*_*A*_. The latter is adjusted such that the lower bound of the hysteresis region extends up to $c_{A}^{*}=0$ as experimentally reported.

**Table 1:** Parameters used in the deterministic and stochastic simulations

**Parameter**	**Description**	**Value**	**Reference**
*K*_*d*1_	dissociation constant of LuxR to A	100 *nM*	[41]
$k_{1}^{-}$	unbinding rate of LuxR to A	10 *min*^−1^	estimated
*K*_*d*2_	dissociation constant of *LuxR*·*AI* dimerization	20 *nM*	fitted
$K_{2}^{-}$	dissociation rate of dimer (*LuxR*·*AI*)_2_	1 *min*^−1^	estimated
*k*_*A*_	synthesis rate of A by LuxI	0.04 *min*^−1^	fitted
*K*_*dlux*_	dissociation constant of (*LuxR*·*AI*)_2_ to the lux promoter	200 *nM*	fitted
$k_{\text{lux}}^{-}$	dissociation rate of (*LuxR*·*AI*)_2_ to the lux promoter	10 *min*^−1^	estimated
*b*	burst size	20	[38]
*k*_*R*_	transcription rate of *luxR*	200/*bmin*^−1^	fitted
*k*_*I*_	transcription rate of *luxI*	50/*bmin*^−1^	fitted
*p*_*R*_	translation rate of *luxR* mRNA	*bd*_*mR*_*min*^−1^	
*p*_*I*_	translation rate of *luxI* mRNA	*bd*_*mI*_*min*^−1^	
*α*_*R*_	ratio between unactivated and activated rate of expression of *luxR*	0.001	fitted
*α*_*I*_	ratio between unactivated and activated rate of expression of *luxI*	0.01	fitted
*d*_*A*_	degradation rate of *A* (same inside and outside the cell)	0.001 *min*^−1^	[42]
$d_{C_{2}}$	degradation rate of (*LuxR*·*AI*)_2_	0.002 *min*^−1^	estimated
*d*_*C*_	degradation rate of *LuxR*·*AI*	0.002 *min*^−1^	estimated
*d*_*R*_	degradation rate of LuxR	0.002 *min*^−1^	estimated
*d*_*I*_	degradation rate of LuxI	0.01 *min*^−1^	estimated
*d*_*mR*_	degradation rate of *luxR* mRNA	0.347 *min*^−1^	[43]
*d*_*mI*_	degradation rate of *luxI* mRNA	0.347 *min*^−1^	[43]
*D*	effective diffusion rate of *A* through the cell membrane	10 *min*^−1^	[44]
*τ*	cell cycle duration (doubling time) in RM/succinate at 30 C	45 *min*	[10]
*λ*	relative weight between the det./sto. components of the cell cycle	0.8	[33,45]
*V*_0_	cell volume at the beginning of cell cycle	1.5 *μm*^3^	[46]
*V*_*tot*_	total cell culture volume	2·10^−4^*μl*	

### First passage time analysis

The mean first passage time at a given autoinducer concentration quantifies the average time that a cell takes to get activated or deactivated. For computing the first passage time in transitions, from low (high) to high (low) state, we take a single cell at the low (high) state and follow its dynamics until the GFP expression level reaches the high (low) state. We point out that the maximum GFP concentration refers to that of the deterministic simulations. In order to get enough statistics, we repeat this procedure, departing from the same initial condition, 10^3^ times for each concentration of autoinducer.

## Results

### The deterministic model reproduces the experimental observations at the population level

The chemical kinetics formalism leads to a set of ODEs that describes the population average dynamics in terms of the concentration of the different species considered in our model (see Additional file 1: Text S1). As in some experiments [10], we assume that the cell culture grows in an environment where the concentration of the external autoinducer in the medium, $c_{A_{\text{ext}}}$, is kept fixed and under well-stirred conditions. In addition, we implement a dilution protocol that compensates for cell growth and maintains the cell density constant (see Methods). We notice that in some experimental setups, e.g. [10], a periodic dilution protocol is applied for keeping the cell density constant; in our model, we keep the cell density constant by means of a continuous influx and efflux of culture medium, as in a chemostat or microfluidic device.

**Figure 3:**
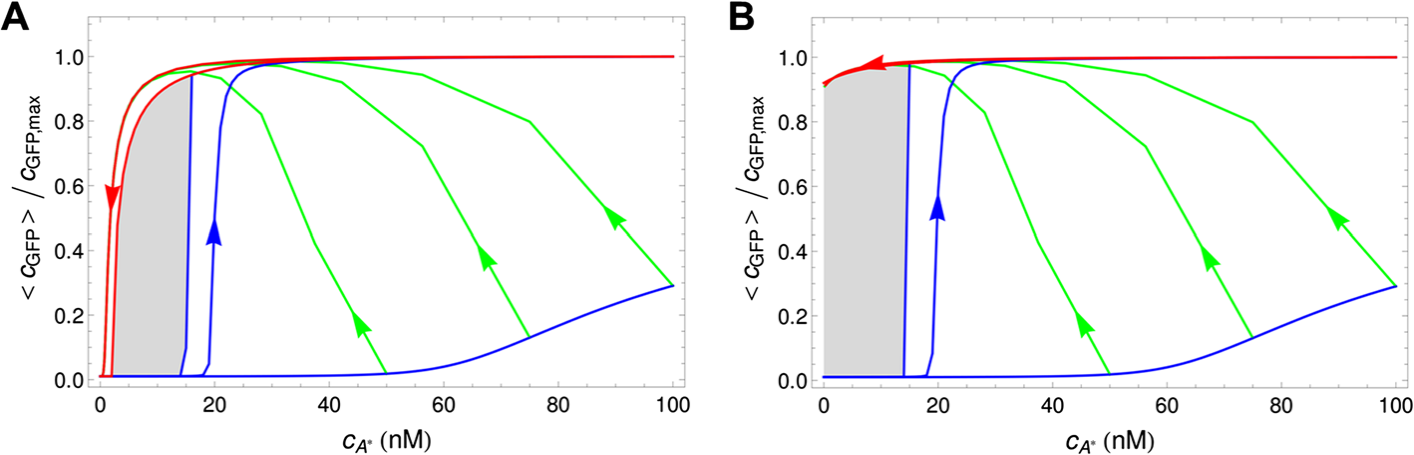
**Response curves to autoinducer induction in the population-average model.***lux01* (**A**) and *lux02* (**B**) operons. The normalized GFP concentration is plotted as a function of the exogenous autoinducer concentration $c_{A}^{*}$: steady-state response for increasing (arrow-free upper blue curve) and decreasing (arrow-free red curve) autoinducer concentration, response under 10 h induction time for increasing (blue curve with arrow) autoinducer concentration, transient response after 2 hours of induction (lower blue curve) from initially non-induced cells, decreasing-concentration trajectories (green curves) for cells weakly induced (2 hours) at $c_{A}^{*}=100\text{nM}$, 75 nM and 50 nM, and decreasing-concentration trajectories (red curve with arrow) for cells fully induced (10 hours) at $c_{A}^{*}=100\text{nM}$. The decreasing-concentration trajectories reduce the value of $c_{A}^{*}$ hourly by 25% (similar to the experiments in [10]). The gray-shaded region between the increasing and decreasing steady-state curves reveals bistability in the range $2\text{nM}<c_{A}^{*}<15\text{nM}$ (*lux01*) and $0\text{nM}<c_{A}^{*}<15\text{nM}$ (*lux02*).

We use the deterministic simulations as a benchmark of the regulatory interactions included in our model and also to fit/estimate some parameters such that the experimental data are reproduced (see [10]). Thus, by integrating numerically the rate equations derived from the population-averaged model, we compute the steady state concentration (induction time 100 hours) of GFP (*lux01*) and LuxI::GFP (*lux02*) as a function of $c_{A}^{*}$. The steady-state induction curves for increasing and decreasing autoinducer concentration of the *lux01* and *lux02* constructs are shown in Figure 3. We are able to reproduce the behaviour of the network at the steady-state, in particular a region of bistability in the range of autoinducer concentration $2\text{nM}<c_{A}^{*}<15$ nM (*lux01*) and $0\text{nM}<c_{A}^{*}<15$ nM (*lux02*). As shown by Williams et al., the *luxR* regulation of the *lux01* operon alone (positive feedback loop) is enough to yield a bistable response. Moreover, expression of LuxI in the *lux02* operon restores the autoinduction loop and extends the lower bound of the hysteresis range to zero concentration of exogenous autoinducer as seen experimentally, indicating that once the operon is fully activated and cells produce their own autoinducer that increases the stability of the high state.

Further simulations to check if the dynamics of our model is compatible with the experimental data refer to the behaviour of the system under non-stationary induction conditions and to the serial dilution protocol of the external medium [10]. As for the first, when cells are induced for 10 h, we observe that the bistability region increases (see Figure 3). As for the second, cells are partially induced at a fixed autoinducer concentration for 2 hours and afterwards the external medium is changed hourly to decrease the concentration of the autoinducer. In this case, the transient response of the cells (Figure 3, green curves) also reproduces the experimental observations. That is, from the point of view of the population average, the deterministic model is not only capable of reproducing the steady-state of the network but also its dynamics. Moreover, in agreement with experiments (see Figure S6 in [10]) our simulations reveal that the temporal scale for reaching a steady-state is much larger than the cell cycle duration. In order to clarify how noise and the induction time modifies the timing for the transition at the single cell level we then perform stochastic simulations.

### The stochastic simulations reveal the interplay between non-stationary effects and noise

Cells are subjected to intrinsic noise at the level of the mRNAs, regulatory proteins, i.e. LuxR and LuxI, and at the level of signalling molecules. In order to analyze the behaviour of individual cells and reveal how noise affects the QS switch, we perform stochastic simulations of a population of growing and dividing cells as described in the Methods section (see Additional file 2: Video S1). The transition of an individual cell from the low to the high state, and the other way around, is intrinsically random and depends, among others, on the levels of autoinducer. Thus, inside a population some cells will jump while others remain in their current state leading to a bimodal phenotypic distribution. We compute the proportion of cells that are below and above a threshold of GFP equal to half-maximum GFP concentration. We consider the distribution of cells to be bimodal when the proportion of cells in either the low or the high state is below 90% and according to this we define the range of autoinducer concentration $\left[c_{A_{b1}^{*}},c_{A_{b2}^{*}}\right]$ for which there is bimodality. For low concentrations of autoinducer, $c_{A}^{*}<c_{A_{b1}^{*}}$, the collective response of the cell population is unactivated, and for high concentrations, $c_{A}^{*}>c_{A_{b2}^{*}}$, such response activates most of the cells leading to a global response of the colony. On the other hand, within the bimodality range, the response is distributed between two subpopulations, thus failing to achieve a global coordination in the colony. In order to characterize this behaviour, we introduce the concept of precision in the QS switch as the inverse of the $c_{A_{*}}$ concentration range for which the cells response distribution (phenotypes), during an induction experiment, is bimodal. That is, the larger the bimodal range, the less precise the switch is in order to generate a global response in the colony. In this regard, we point out that the precision of the switch in a noise-free situation is infinite since all cells achieve global coordination simultaneously.

**Figure 4:**
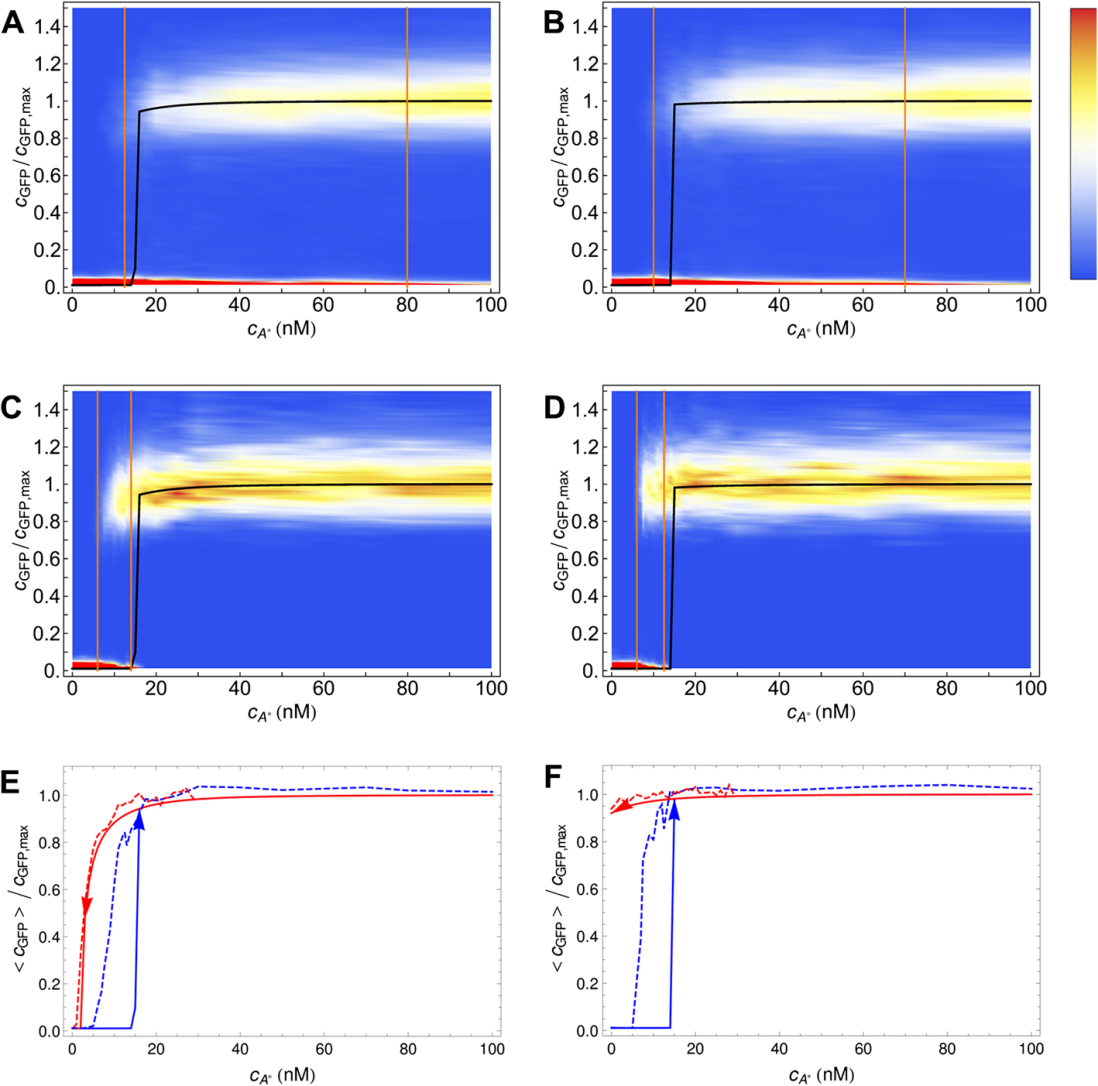
**Cell response distribution to autoinducer induction in the stochastic model.** Cell response probability after 10 hours (top: **A**, **B**) and 100 hours (middle: **C**, **D**) of induction at different autoinducer concentrations for the *lux01* (left: **A**, **C**) and *lux02* (right: **B**, **D**) operons in the stochastic model. The distribution reveals the coexistence of two subpopulations with low and high GFP expression when the cells are induced at intermediate autoinducer concentrations. The region of bistability (precision) is defined by the range of $c_{A}^{*}$ for which the response is bimodal according to the following criterion: the lower/upper limit of the bistable region (orange lines) is defined by the value of $c_{A}^{*}$ for which 90% of the cells are in the low/high state. The black line stands for the concentration of GFP (normalized) as a function of $c_{A}^{*}$ in the deterministic model at the steady state. After 10 hours of induction (top: **A**, **B**) most cells are still in a transient state if $c_{A}^{*}<70\text{nM}$. After 100 hours of induction (middle: **C**, **D**), the bimodality region shrinks and the precision increases. The population average curves of the induction and dilution experiments in the stochastic model (bottom: **E**, **F**, dashed lines) show that the intrinsic noise allows cells to jump to the high state inside the deterministic bistable region. On the other hand, the transition from high to low follows the deterministic path thus indicating that the switching rate in this case is close to zero.

**Figure 5:**
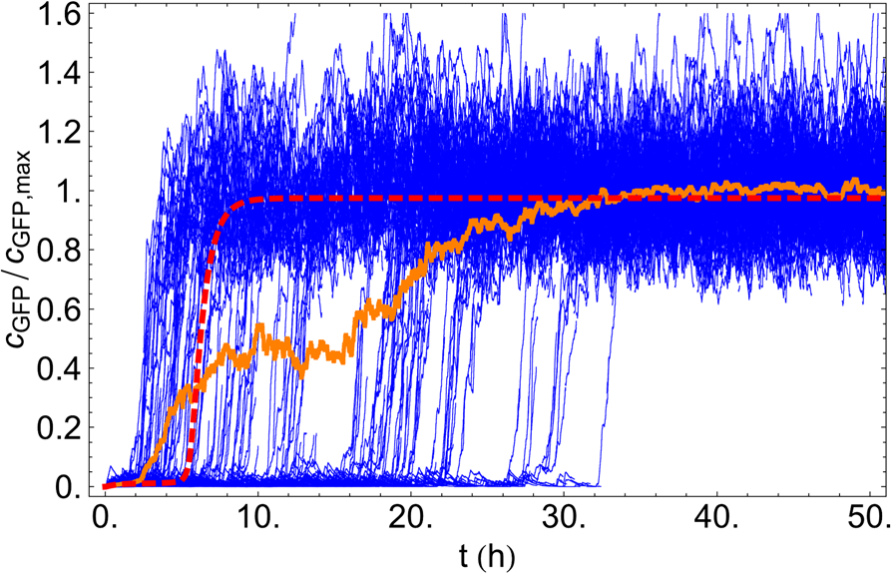
**Individual cell trajectories for autoinducer induction in the stochastic model.** Individual cell trajectories (blue lines), cell population average (orange line) and deterministic solution (red dashed line) for an induction experiment at $c_{A}^{*}=25\text{nM}$ for the *lux01* operon in the stochastic model. Individual cell trajectories show the heterogeneous distribution of cell jumping times. While some cells achieve full induction of the operon before the deterministic case, the global response of the population reaches steady-state at ∼30 hours, slower than the deterministic solution.

Figure 4 shows, by means of a color density plot, the probability of a cell to have a particular GFP expression level after either 10 or 100 hours of induction as a function of $c_{A}^{*}$. In order to gather enough statistics, we average our results over 10 different realizations (i.e. experiments). For a large range of autoinducer concentrations, for both the *lux01* and for the *lux02* operon, the distribution of GFP expression after 10 h of induction is bimodal. As shown, some cells of the colony are induced before the critical concentration of the deterministic model at the steady state (black line). Still, the concentration for which more than 90% of the cells are induced requires up to four times more autoinducer than under deterministic conditions. Thus, on the one hand noise can help cells to get induced at lower autoinducer concentrations but, on the other hand, amplifies the non-stationary effects for achieving global coordination. In order to clarify this interplay between non-stationary and stochastic effects, we perform the same simulations with a larger induction time (100 h). As expected, the precision of the switch increases (10-fold change) and cells achieve global coordination at (*lux01*) or before (*lux02*) the critical deterministic concentration. Note that in all cases noise induces a significant variability in terms of the GFP expression levels in the high state compared to that of the low state (see also Figure 5). The variability introduced in the colony response by the fluctuations with respect to the deterministic approach can also be observed in experiments under weak inducing conditions where the autoinducer concentration is periodically decreased (see Additional file 4: Figure S2).

**Figure 6:**
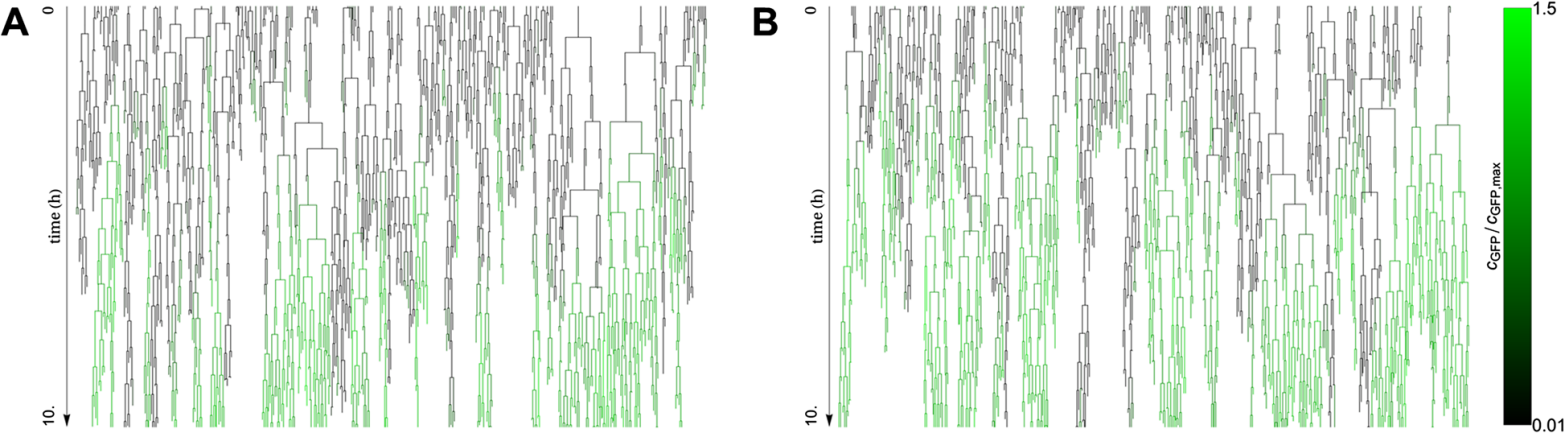
**Lineage tree of an induced population of cells in the stochastic model.** Linage tree of a population of cells induced at a fixed autoinducer concentration $c_{A}^{*}=50\text{nM}$ for the *lux01* operon (left) and the *lux02* operon (right). Vertical lines represent individual cells and horizontal lines cell division events. The color of the lines is proportional to the normalized GFP expression. The initial number of cells is 100 and is kept constant during the experiment by “deleting” cells at random every time a cell divides (truncated vertical lines). The lineage tree shows how the state of the cell is transmitted over generations and reveals that once the operon is activated the transition is “irreversible”.

The heterogeneity in terms of the jumping statistics is revealed in Figure 5 where we plot individual trajectories for the *lux01* operon as a function of time at $c_{A}^{*}=25\;\text{nM}$ over a period of 50 hours. Some cells become induced after 3 hours, while others need ∼10 times more induction time to reach the high state. At this concentration of autoinducer all cells have eventually reached the high state after ∼30 hours of induction. Importantly, we do not observe that cells jump back (see Discussion). That is, while there is variability over the colony in regards of the switching time, once the transition occurs the cell remains in the new state that is sustained over generations as seen in Figure 6. Therefore, over the typical timescale of an experiment (10 to 50 hours), the behaviour of the QS switch is highly dynamic and the precision of the switch is a transient quantity that crucially depends on the duration of induction.

As expected the intrinsic noise decreases the precision of the QS switch with respect to the deterministic case. Still, noise helps cells to become activated before the critical concentration of a fluctuations-free system under all induction conditions. Moreover, in steady-state conditions the high state is globally achieved before the critical deterministic concentration. This phenomenon is recapitulated in Figure 4 (bottom) where we plot the population average response for the induction and dilution experiments at steady-state (100 h induction) for both the deterministic and stochastic models. Notice that the dilution curves of the stochastic model are similar to that of the deterministic model; however, the average transition to the high state occurs at a lower autoinducer concentration due to intrinsic fluctuations.

### The features of the QS switch depends on the transcriptional noise of LuxR

**Figure 7:**
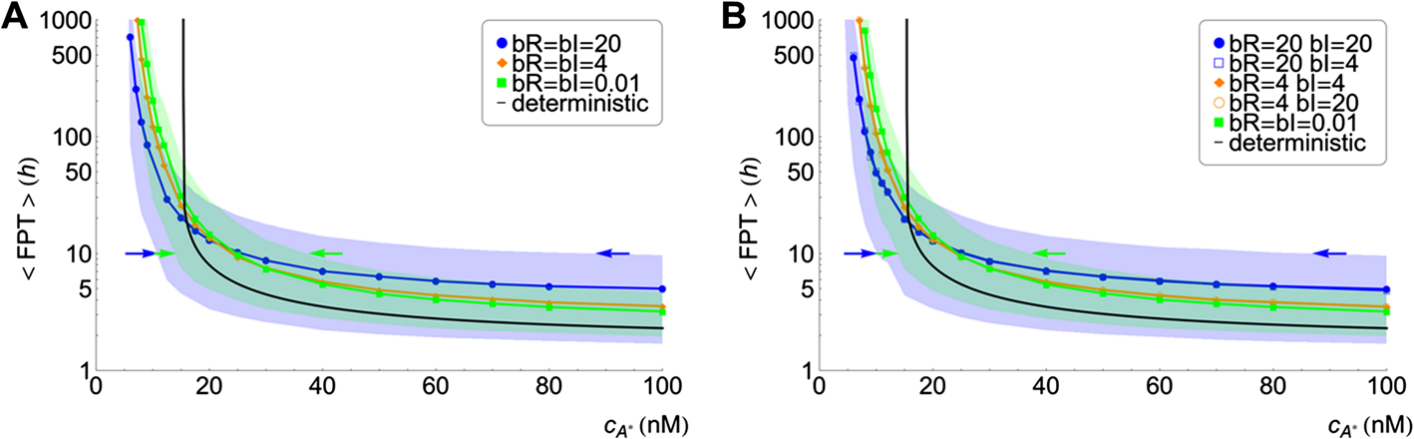
**Mean first passage time of cell activation for different burst size values.** Mean first passage time of cell activation as a function of autoinducer concentration for different values of the burst size for LuxR (*b*_*R*_) and LuxI (*b*_*I*_) and for the deterministic solution: (**A**) low to high transition MFPT in the *lux01* operon, (**B**) low to high transition MFPT in the *lux02* operon. The lower (upper) limit of the shaded regions is the 10% (90%) quantile curve of the distribution of FPT for the cases *b*_*R*_=*b*_*I*_=20 (blue shaded region) and *b*_*R*_=*b*_*I*_=0.01 (green shaded region). The MFPT reveals a non-trivial behaviour: for low autoinducer concentration noise helps cells to jump quicker to the high state, while for high autoinducer concentration noise slows down the cells activation (see text). Intersections of the quantile 10% and quantile 90% curves with a horizontal line at *t*=10h indicate the autoinducer concentration for which 10% of cell trajectories have jumped to the high state (left arrow) and the concentration for which 90% of cell trajectories have been activated (right arrow). The precision after 10h of induction (inversely proportional to the width of the region delimited by the arrows), increases when decreasing the noise in LuxR (see text). Note that in the case of the *lux01* operon, we only change the value of *b*_*R*_since GFP does not contribute to the activation process.

For the same concentration of the external autoinducer, the stochastic dynamics of the regulatory network arises from the noise at the level of LuxI and LuxR. We now analyze the individual contribution of those by modulating the burst size of LuxR and LuxI, *b*_*R*_and *b*_*I*_respectively. We notice that the burst size modulates the stochasticity levels while maintaining the average protein copy numbers. Additional file 5: Figure S3 illustrates the effect of changing the burst size by showing individual trajectories of the chemical species obtained for large and small values of this quantity at low and high concentrations of the external autoinducer. In this regard, insight about the activation process can be obtained by computing the mean first passage time (MFPT) for transitions between the low and the high state. Figure 7 shows this quantity as a function of $c_{A}^{*}$ and for different values of the burst size of LuxR and LuxI. For the sake of comparison, we also compute the MFPT for the deterministic solution. We note that in that case, the MFPT inside the bistable region is infinite, since the deterministic system cannot spontaneously jump from one stable state to the other. Our results indicate that changing the burst size of LuxI does not modify the mean first passage time whereas changing the transcriptional noise at the level of LuxR modifies the jumping statistics. Moreover, our results reveal a non-trivial behaviour of the MFPT as a function of the concentration of the autoinducer. On one hand, with respect to the activation dynamics, when $c_{A}^{*}$ is below ∼25 nM, an increase in LuxR noise decreases the mean time of the activation. That is, LuxR noise helps cells to get the initial activation quicker. On the other hand, above ∼25 nM of autoinducer concentration, the effect is the opposite: an increase in LuxR noise increases the mean jumping time thus slowing down the full cell activation.

**Figure 8:**
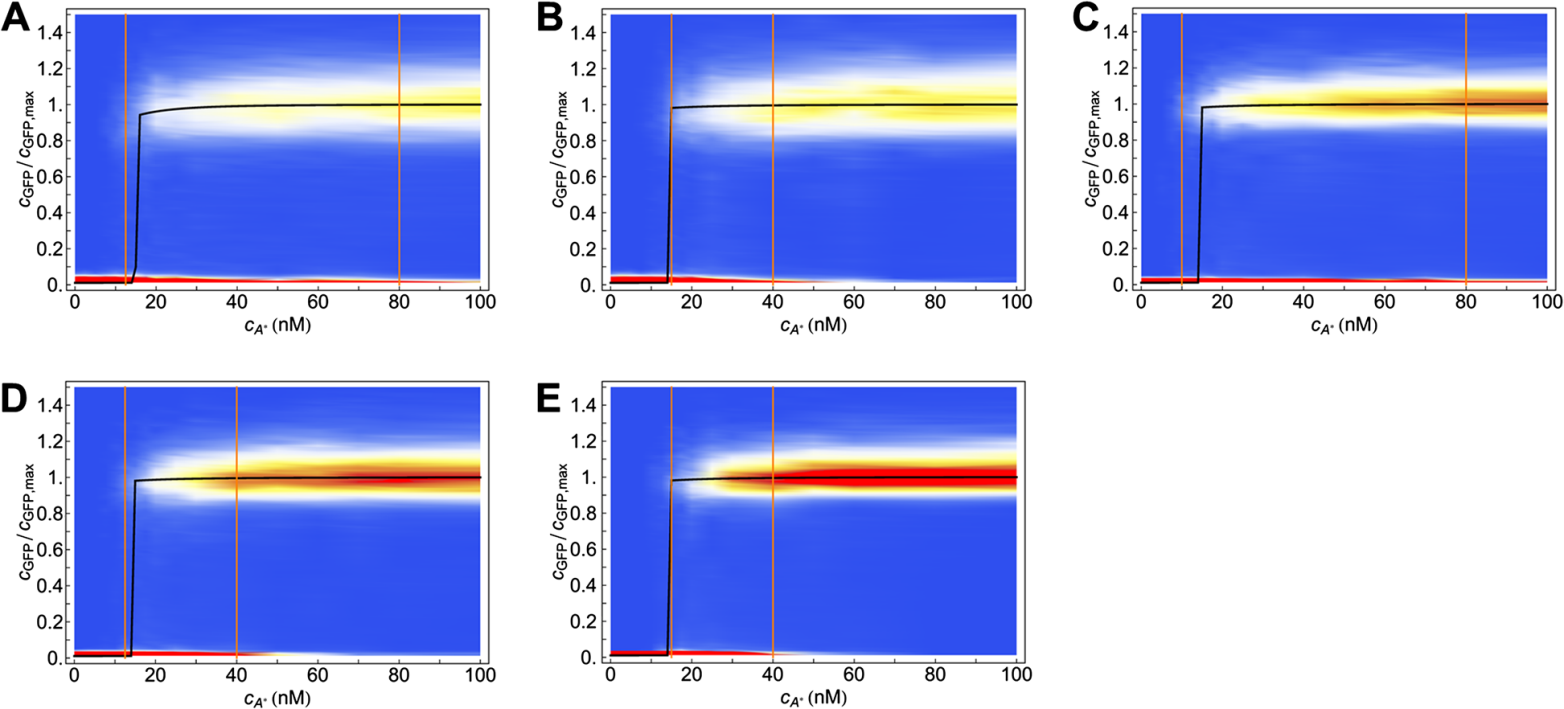
**Cell response distribution in the transient regime for different burst size values.** Cell response distribution (jumping probability) after 10 hours of induction (transient state) at different autoinducer concentrations for the *lux02* operon in the stochastic model and different burst sizes. Burst size values (**A**) *b*_*R*_=*b*_*I*_=20 (**B**) *b*_*R*_=4,b_*I*_=20 (**C**) *b*_*R*_=20,b_*I*_=4 (**D**) *b*_*R*_=*b*_*I*_=4 (**E**) *b*_*R*_=*b*_*I*_=0.01. Width of bistable region: (**A**) = 60 nM (**B**) 25 nM (**C**) 70 nM (**D**) 27.5 nM (**E**) 25 nM. The black line stands for the concentration of GFP (normalized) as a function of $c_{A}^{*}$ in the deterministic model at the steady state.

**Figure 9:**
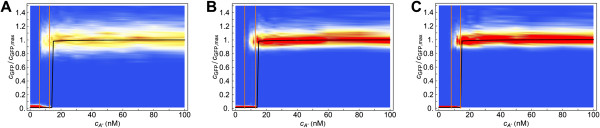
**Cell response distribution at the steady-state for different burst size values.** Cell response distribution at the steady-state (100 h induction), at different autoinducer concentrations for the *lux02* operon in the stochastic model for different burst size values: (**A**) *b*_*R*_=*b*_*I*_=20 (**B**) *b*_*R*_=*b*_*I*_=4 (**C**) *b*_*R*_=*b*_*I*_=0.01. The probability density of getting a particular GFP expression level is indicated by means of a density plot. The width of bistable region barely depends on the stochasticity levels, ≈7 nM. The black line stands for the concentration of GFP (normalized) as a function of $c_{A}^{*}$ in the deterministic model at the steady state.

We observe these effects both for the *lux01* and *lux02* operons. Surprisingly, when the autoinducer concentration is above the critical concentration of the deterministic system, $c_{A}^{*}\approx 20$ nM, the stochastic system always takes more time to get activated than the deterministic case. By computing additional properties of the first passage time probability density we also clarify the behaviour of the precision depending on the induction time. In particular, we compute the times *t*_*low*_ and *t*_*high*_ for which, at a given $c_{A}^{*}$ concentration, the probabilities of finding a FPT<*t*_*low*_and a FPT>*t*_*high*_are 10%, i.e. the 10% and 90% quantiles respectively. The shadings in Figure 7 delimit these regions for the cases *b*_*R*_=*b*_*I*_=20 and *b*_*R*_=*b*_*I*_=0.01. The precision of the switch after *n* hours of induction, is directly related to the width of the shaded region at 〈FPT〉=*n* h: at any given time, this width indicates which is the minimal concentration of autoinducer for getting 10% of cells already activated and also the concentration beyond which more than 90% of cells have been activated. Thus, in agreement with Figure 4, the induction time clearly modifies the precision: it increases (the width decreases) as the induction time becomes larger. Moreover, note that as the LuxR noise weakens the precision increases. Figure 8 recapitulates some of these results. There we show the GFP expression probability for the *lux02* operon after 10 hours of induction for different values of the burst size *b*_*R*_ and *b*_*I*_. Notice that the region of bimodality does not vary when changing the burst size for LuxI. However, decreasing the burst size in LuxR reduces the region of bimodality thus increasing the precision of the switch. Furthermore, the noise at the level of LuxR helps some cells to become activated at lower concentration levels of the autoinducer. Once more, this phenomenon does not depend on the levels of transcriptional noise of LuxI. That is, while the global coordination increases as the transcriptional noise of LuxR decreases, more concentration of the autoinducer is required to start activating cells. Figure 7 also suggests that the sensitivity of the precision as a function of the induction time and/or as a function of the stochasticity levels get diminished after ∼30 hours since the width of the shaded region barely varies. Figure 9 points towards that direction: under long induction time conditions (100 h) the precision of the switch remains constant regardless the value of the burst size. All together, these results indicate an interesting and counterintuitive role of the transcriptional noise of LuxR in terms of the biological function of the QS switch.

## Discussion

The response of bacterial colonies driven by the QS signalling mechanism under noisy conditions has been addressed, in a broad sense, by different authors. In particular, the characterization of the collective response as a synchronization phenomenon where the phenotypic variations can be generically predicted has been proposed [47]. However, this approach requires gene regulatory interactions controlling the QS switch that do not induce bistability and lead to a monostable behaviour, e.g. negative feedback loops [48]. Our study focus on strains that display, as the wild-type LuxI/LuxR system do, bistability and, consequently, an alternative method to quantify the phenotypic variability induced by noise was needed, i.e. the precision concept. Moreover, previous works assume stationary conditions and disregard the role of the cell cycle duration. Herein, in agreement with experimental results, we have shown that the time for reaching a steady expression rate is much larger than the cell cycle duration (see [10]). As a result, we have revealed that the interplay between non-stationary and stochastic effects is key for understanding the global response of the colony and the phenotypic variability. Finally, we have shown that the intrinsic noise is able to stabilize a particular phenotypic state. This effect, namely the fluctuations inducing a slowing down in the activation of the cells, emerges because noise extends the bistable region compared to the deterministic system. While such a noise-induced phenomenon has been characterized in population models [49] and, more recently, in theoretical studies on bistable switches [18], to the best of our knowledge, this is the first time that is reported in the context of QS systems. All in all, from the viewpoint of the comprehension of how noisy inputs may condition phenotypic variability in bacterial colonies, our study introduces a number of advances.

Herein, we have characterized how the precision of the QS switch depends on the stochasticity levels and, importantly, elucidated which noisy component of the LuxI/LuxR regulatory network drives the observed phenomenology. Thus, we have found that under non-stationary conditions, LuxR controls the phenotypic variability and that changing the noise intensity at the level of LuxI has no effect on the precision of the switch. A plausible explanation for this reads as follows. The fluctuations at the level of LuxI are transmitted to the autoinducer. However, the diffusion mechanism rapidly averages out the stochasticity levels of the latter. This is not possible for LuxR which is kept within the cell. As a consequence the amount of activation complex, that is ultimately the responsible for the activation, is driven by the fluctuations of LuxR but not by those of LuxI.

Recent experimental work has measured the bioluminescence levels of individual *V. fischeri* cells at fixed autoinducer concentration [13]. In agreement with our results, the authors observed that cells differed widely in terms of their activation time and luminescence distribution. Interestingly, other experiments have revealed the presence of additional regulatory interactions for controlling the LuxR noise levels. For example, C8HSL molecules, a second QS signal in *V. fischeri*, has been suggested to reduce the noise in bioluminescence output of the cells at low autoinducer concentrations [50]. In the same direction, in *V. harveyi*, the number of LuxR dimers is tightly regulated indicating a control over LuxR intrinsic noise [51]. In fact, wild-type *V. harveyi* strains have two negative feedback loops that repress the production of LuxR [52] and this kind of regulatory circuit is known to reduce noise levels [53]. In this context, our results provide a feasible explanation for the network structure in wild-type strains: since noise in LuxR controls the phenotypic variability of the LuxR/LuxI QS systems, bacteria have evolved mechanisms to control its noise levels. An additional argument in this regard arises from our results about the deactivation of cells: once they are fully induced we do not observe reversibility of the phenotype (FPT larger than 100 h). First, these results are in agreement with other switching systems as the gallactose signalling network in yeast [54] and with theoretical results that explain the asymmetric switching dynamics due to stochastic effects [18]. Second, they reveal the importance of additional interactions that regulate negatively *luxR* in wild-type strains and indicates that synthetic strains as *lux01* and *lux02* summarize many features of the wild-type operon during the activation process but fail to capture some of dynamical aspects of the deactivation phenomenon.

**Figure 10:**
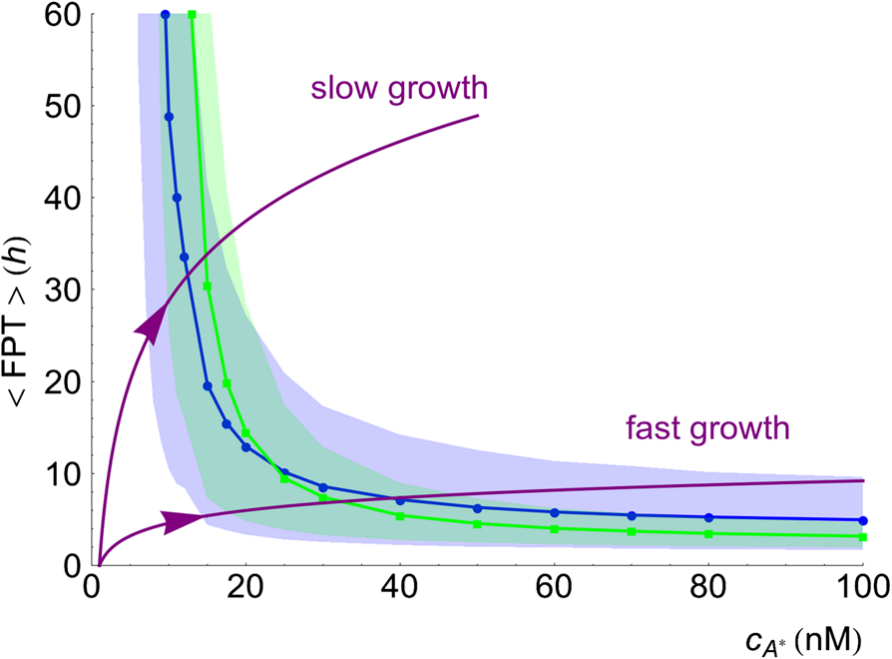
**The growth rate conditions the phenotypic variability.** In the context of a growing colony, the autoinducer concentration increases as the colony does: purple lines show schematically two exponential growth conditions for the autoinducer concentration as a function of time. Our results on the MFPT, valid at fixed autoinducer concentrations, can be extrapolated, qualitatively, to the case of increasing autoinducer levels. Fast growth results in a large cell variability and large critical colony size for achieving a global response, while slow growth produces reduced cell variability and a smaller critical population size. Increasing fluctuations in LuxR have two opposite effects: in the slow growth case, increasing the noise (blue curves: *b*_*R*_=20; green curves: *b*_*R*_=0.01;) decreases the critical population size while hardly changing the variability, in the fast growth case, increasing noise increases the critical population size and increases greatly the variability.

Finally, our simulations indicate that non-stationary effects are essential during the activation of the QS response. While speculative, these results can be extrapolated to growing colonies where the cell density is not kept constant. A good supply of nutrients implies short induction times since the concentration of autoinducer will quickly grow (exponentially) as the population size does. According to our results, this fast growing condition decreases the precision of the switch and, consequently, promotes variability at the population level (see Figure 10). In addition, the full collective activation of the system would require a large population size. On the other hand, if the colony grows in a poor nutrient environment, the system will have time to reach a steady-state more easily and the precision would increase. Hence, the variability would be diminished, and full activation would require smaller colony sizes. Most phenotypic changes induced by the QS mechanism refer to bacterial strategies for survival and/or colonization. In this context, our results suggest that both the QS activation threshold and the phenotypic variability might depend on the growth rate of the colony and, as a consequence, on the environmental conditions. This is in agreement with recent studies that show that the collective response of a population of cells depends not only on the underlying genetic circuit and the environmental signals, but also on the speed of variation of these signals [55].

## Conclusions

Herein we have introduced deterministic and stochastic modelling approaches for describing the core functionality of the LuxI/LuxR regulatory network in quorum sensing systems. We have focused on synthetic constructs, *lux01* and *lux02*, that reproduce the behaviour of the wild-type system and allow for controlled experiments that have provided quantification of the activation process [10]. The deterministic approach has allowed us to estimate different parameters of the model and reproduce the switch-like behaviour of the QS network. Thus, our simulations reveal that the interplay between non-stationary and stochastic effects are key and that, for an extended range of autoinducer concentrations, a bimodal phenotypic variability develops such that cells fail to produce a global response. In this context we have introduced the concept of precision of the QS switch, as the inverse of the width of the bimodal phenotypic region.

By computing the statistics of the activation dynamics of cells, we have shown that the QS precision depends on the gene expression noise at the level of LuxR and is independent from that of LuxI. Our results, together with the experimental evidences on LuxR regulation in wild-type species, suggest that the noise at the level of LuxR controls the phenotypic variability of the LuxR/LuxI QS systems and that bacteria have evolved to control its intensity. In addition, the robust stabilization of the phenotype once is fully induced indicates that, albeit synthetic strains as *lux01* and *lux02* summarize many features of the wild-type operon during the activation process, they fail to capture crucial aspects of the deactivation phenomenon.

Most insight in regards of the effect of LuxR noise on the dynamics of cell activation is given by the study of the mean first passage time (MFPT). In terms of the timing of activation, we have observed two opposite effects depending on the control parameter $c_{A}^{*}$: for $c_{A}^{*}≾20$ nM, the larger the noise in LuxR, the quicker the cells become activated, while for $c_{A}^{*}≿20$ nM, we observe the opposite effect and noise slows down cell activation. We suggest that this effect can be explained by the stochastic stabilization of the low state. Moreover, the calculation of additional properties of the statistics of the first passage time have allowed us to relate the concept of precision of the switch with the variability of the FPT by estimating the 10% and 90% quantiles.

In summary, our results indicate that in bacterial colonies driven by the QS mechanism there is a trade-off between the activation onset and a global response due to non-stationary and stochastic effects. On one hand, large levels of noise at the level of LuxR imply that cells require smaller autoinducer levels for achieving an activation onset but, at the same time, a global response requires a substantial autoinducer concentration. On the other hand, if the LuxR noise levels are small, the activation onset is shifted toward larger values of the autoinducer concentration but the global response is achieved for smaller concentration values. Our study could be useful for Synthetic Biology approaches that exploit the QS mechanism. The fact that some important features of the QS mechanism, e.g. precision, rely on the burst size of one component, opens the door to modifications of the LuxI/LuxR operon for regulating the response depending on the problem under consideration. Finally, further research is needed about the general validity and applicability on the noise-induced stabilization phenomenon of particular phenotypic states in other gene regulatory systems beyond the QS mechanism. Work in that direction is in progress.

## Competing interests

The authors declare that they have no competing interests.

## Authors’ contributions

MW and JB designed the experiments. MW carried out the simulations. MW and JB analyzed the data. All authors read and approved the final manuscript.

## Supplementary Material

Additional file 1 **Text S1.** Chemical equations for the deterministic model. Click here for file

Additional file 2 **Video S1.** Movie of the stochastic simulation. Movie of the stochastic simulation for the *lux02* operon, 10 h of induction at $c_{A}^{*}=50\text{nM}$, burst size *b*_*R*_=*b*_*I*_=4. Cells are modelled as individual compartments containing a copy of the LuxR/LuxI regulatory network. The Gillespie algorithm (see text for details) is used to integrate the stochastic dynamics of the whole system of cells. Cell growth and division is explicitly taken into account as well as a certain degree of stochasticity in the cell cycle duration. Cells movement is purely aesthetic since we do not include any spatial effects in our model and consider a well-mixed environment. The number of cells (*N*=100) is maintained constant by removing one cell at random each time a cell divides. Click here for file

Additional file 3 **Figure S1.** Intra and extracellular autoinducer as a function of exogeneous autoinducer concentration. Response curves to autoinducer induction for *lux01* (**A**, **C** and **E**) and *lux02* (**B**, **D** and **E**) operons. Total autoinducer concentration $c_{A_{\text{tot}}}$ in the external volume and in the cells (**A** and **B**), intracellular concentration *c*_*A*_(**C** and **D**), and extracellular concentration $c_{A_{\text{ext}}}$ (**E** and **F**), as a function of the exogenous autoinducer concentration, $c_{A}^{*}$, in the deterministic model. All graphs represent the steady-state response for increasing (blue curve) and decreasing (red curve) autoinducer concentrations. The exogeneous autoinducer concentration $c_{A}^{*}$ controls the autoinducer concentration in the medium by means of an influx and an efflux (see main text). Upon activation of the operon, LuxR is produced at high levels, thus sequestering autoinducer molecules inside the cells. The bound form of autoinducer cannot diffuse out of the cell and is therefore not subjected to the influx and efflux. This explains why the total concentration of autoinducer in the system, $c_{A_{\text{tot}}}=\frac{1}{V_{\text{tot}}}\left[V_{\text{cell}}\left(c_{A}+c_{\text{luxR}\cdot A}+c_{{\left(\text{luxR}\cdot A\right)}_{2}}+c_{\text{DNA}\cdot {\left(\text{luxR}\cdot A\right)}_{2}}\right)+V_{\text{ext}}c_{A_{\text{ext}}}\right]$ is slightly larger than $c_{A}^{*}$, when the operon is activated. For the same reason, the free form of autoinducer, both in the cell and in the medium, is slightly smaller. Click here for file

Additional file 4 **Figure S2.** Cell response distribution during decreasing-concentration trajectories. Cell response distribution for decreasing-concentration trajectories for *lux01* (left) and *lux02* (right) strains in the stochastic model. Cells are initially induced at $c_{A}^{*}=100\text{nM}$ for 2 hours. The concentration of exogenous autoinducer $c_{A}^{*}$ is then hourly decreased in order to simulate the experiments (see [10]). The cell distribution reveals the variety of cell trajectories in comparison to the deterministic population average solution (green line). The cells jump to the high state for a wide range of times and autoinducer concentrations. Note also that fluctuations leads to a stabilization of the low state with respect to the deterministic solution. Click here for file

Additional file 5 **Figure S3.** Trajectory of chemical species in individual cells. Trajectory of chemical species LuxR mRNA (mR), LuxR, LuxI, intracellular autoinducer (AI), regulatory complex (*LuxR*·*AI*)_2_(AL2) and promoter bound to complex (P10), in an individual cell for the following control parameter and burst size values: (A) $c_{A}^{*}=15\;\text{nM},\;b_{R}=b_{I}=20$, (B) $c_{A}^{*}=50\;\text{nM},\;b_{R}=b_{I}=20$, (C) $c_{A}^{*}=15\;\text{nM},\;b_{R}=b_{I}=0.01$, (D) $c_{A}^{*}=50\;\text{nM},\;b_{R}=b_{I}=0.01$. Click here for file
