# Supplementary material for: Dynamics of the quorum sensing switch: stochastic and non-stationary effects
Source: BMC Syst Biol. 2013 Jan 16;7:6. doi: 10.1186/1752-0509-7-6 (PMC3614889; doi:10.1186/1752-0509-7-6)
Supplement: Additional file 1 — Text S1. Chemical equations for the deterministic model. [file 1752-0509-7-6-S1.pdf]

Supplementary Information, Text S1

Chemical rate equations for the deterministic model.

$$\dot{c}_{luxI::gfp} = p_I c_{mRNA_{luxI::gfp}} - \left(\frac{ln(2)}{\tau} + d_I\right) c_{luxI::gfp} \tag{1}$$

$$\dot{c}_{luxR} = -\frac{k_1^-}{K_{d1}} c_A c_{luxR} + k_1^- c_{luxR\cdot A} + p_R c_{mRNA_{luxR}} - \left(\frac{ln(2)}{\tau} + d_R\right) c_{luxR} \tag{2}$$

$$\dot{c}_{mRNA_{luxI::gfp}} = \alpha_I k_I c_{DNA} + k_I c_{DNA\cdot(luxR\cdot A)_2} - \left(\frac{ln(2)}{\tau} + d_{mI}\right) c_{mRNA_{luxI::gfp}} \tag{3}$$

$$\dot{c}_{mRNA_{luxR}} = \alpha_R k_R c_{DNA} + k_R c_{DNA\cdot(luxR\cdot A)_2} - \left(\frac{ln(2)}{\tau} + d_{mR}\right) c_{mRNA_{luxR}} \tag{4}$$

$$\dot{c}_{luxR\cdot A} = -k_1^- c_{luxR\cdot A} + \frac{k_1^-}{K_{d1}} c_A c_{luxR} - 2\frac{k_2^-}{K_{d2}} \left[c_{luxR\cdot A}\right]^2 + 2k_2^- c_{(luxR\cdot A)_2} \tag{5}$$

$$- \left(\frac{ln(2)}{\tau} + d_C\right) c_{luxR\cdot A} \tag{6}$$

$$\dot{c}_{(luxR\cdot A)_2} = \frac{k_2^-}{K_{d2}} \left[c_{luxR\cdot A}\right]^2 - k_2^- c_{(luxR\cdot A)_2} - \frac{k_{lux}^-}{K_{dlux}} c_{(luxR\cdot A)_2} c_{DNA} + k_{lux}^- c_{DNA\cdot(luxR\cdot A)_2} \tag{7}$$

$$- \left(\frac{ln(2)}{\tau} + d_{C_2}\right) c_{(luxR\cdot A)_2} \tag{8}$$

$$\dot{c}_{DNA} = -\frac{k_{lux}^-}{K_{dlux}} c_{(luxR\cdot A)_2} c_{DNA} + k_{lux}^- c_{DNA\cdot(luxR\cdot A)_2} + \frac{ln(2)}{\tau} \left(c_{DNA} + c_{DNA\cdot(luxR\cdot A)_2}\right) \tag{9}$$

$$- \frac{ln(2)}{\tau} c_{DNA} \tag{10}$$

$$\dot{c}_{DNA\cdot(luxR\cdot A)_2} = \frac{k_{lux}^-}{K_{dlux}} c_{(luxR\cdot A)_2} c_{DNA} - k_{lux}^- c_{DNA\cdot(luxR\cdot A)_2} - \frac{ln(2)}{\tau} c_{DNA\cdot(luxR\cdot A)_2} \tag{11}$$

$$\dot{c}_A = k_1^- c_{luxR\cdot A} - \frac{k_1^-}{K_{d1}} c_A c_{luxR} + k_A c_{luxI::gfp} + D\left(c_{A_{ext}} - c_A\right) \tag{12}$$

$$- \left(\frac{ln(2)}{\tau} + d_A\right) c_A \tag{13}$$

$$\dot{c}_{A_{ext}} = rD\left(c_A - c_{A_{ext}}\right) + \gamma \frac{V_{tot}}{V_{ext}} c_{A^*} - (\gamma + d_A) c_{A_{ext}} \tag{14}$$
