# Supplementary figures and images for: Dynamics of the quorum sensing switch: stochastic and non-stationary effects
Source: BMC Syst Biol. 2013 Jan 16;7:6. doi: 10.1186/1752-0509-7-6 (PMC3614889; doi:10.1186/1752-0509-7-6)

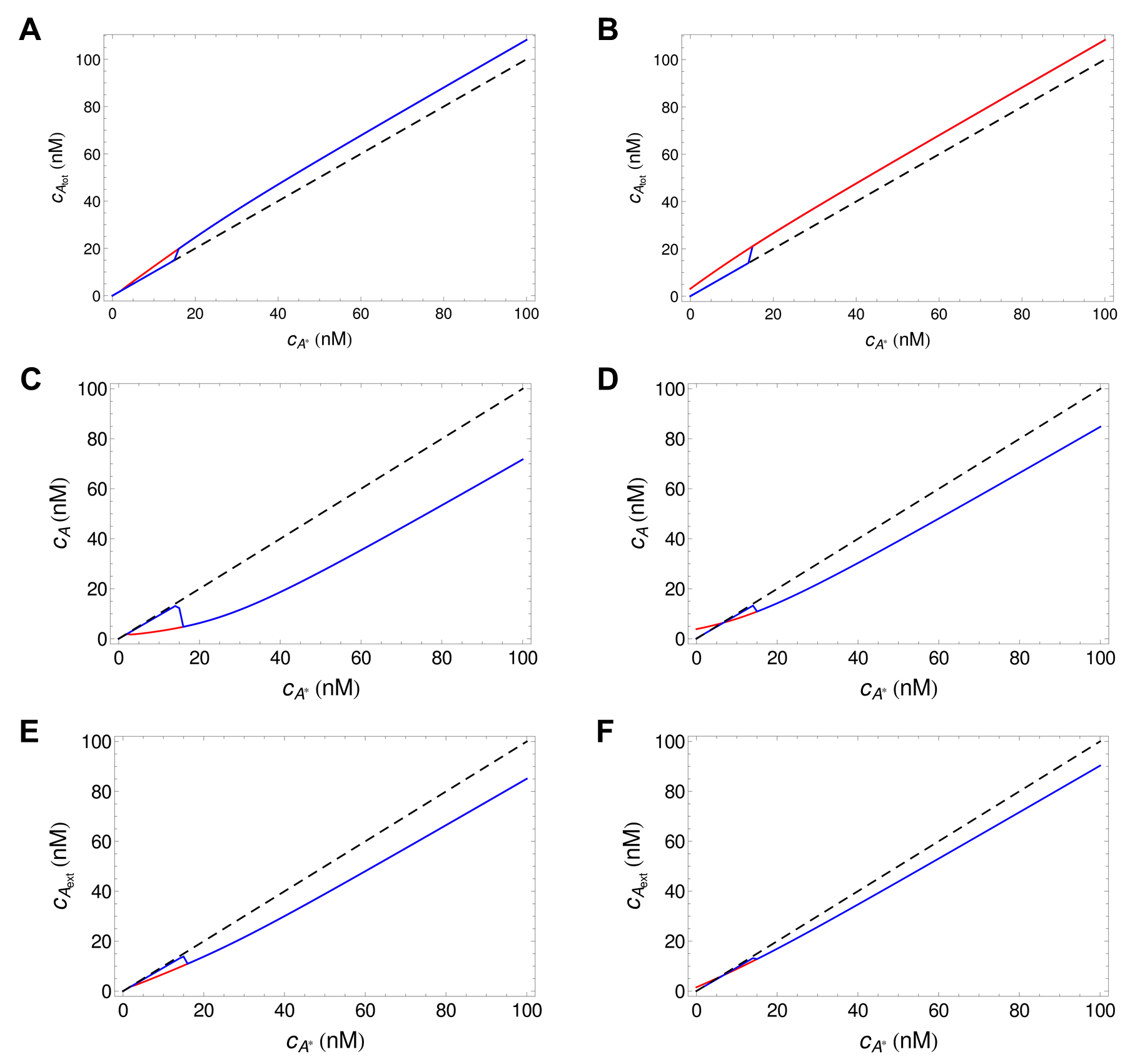

Supplement: Additional file 3 — Figure S1. Intra and extracellular autoinducer as a function of exogeneous autoinducer concentration. Response curves to autoinducer induction for lux01 (A, C and E) and lux02 (B, D and E) operons. Total autoinducer concentration cAtot in the external volume and in the cells (A and B), intracellular concentration cA(C and D), and extracellular concentration cAext (E and F), as a function of the exogenous autoinducer concentration, cA∗, in the deterministic model. All graphs represent the steady-state response for increasing (blue curve) and decreasing (red curve) autoinducer concentrations. The exogeneous autoinducer concentration cA∗ controls the autoinducer concentration in the medium by means of an influx and an efflux (see main text). Upon activation of the operon, LuxR is produced at high levels, thus sequestering autoinducer molecules inside the cells. The bound form of autoinducer cannot diffuse out of the cell and is therefore not subjected to the influx and efflux. This explains why the total concentration of autoinducer in the system, cAtot=1VtotVcellcA+cluxR·A+cluxR·A2+cDNA·luxR·A2+VextcAext is slightly larger than cA∗, when the operon is activated. For the same reason, the free form of autoinducer, both in the cell and in the medium, is slightly smaller. [file 1752-0509-7-6-S3.png]

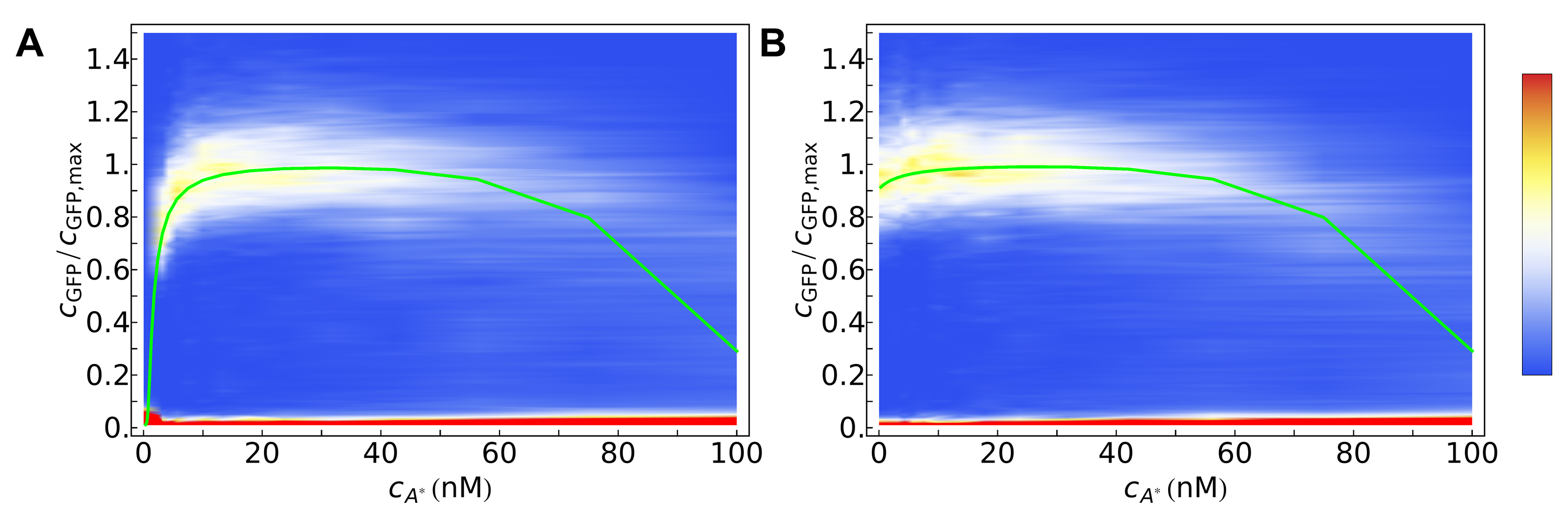

Supplement: Additional file 4 — Figure S2. Cell response distribution during decreasing-concentration trajectories. Cell response distribution for decreasing-concentration trajectories for lux01 (left) and lux02 (right) strains in the stochastic model. Cells are initially induced at cA∗=100nM for 2 hours. The concentration of exogenous autoinducer cA∗ is then hourly decreased in order to simulate the experiments (see [10]). The cell distribution reveals the variety of cell trajectories in comparison to the deterministic population average solution (green line). The cells jump to the high state for a wide range of times and autoinducer concentrations. Note also that fluctuations leads to a stabilization of the low state with respect to the deterministic solution. [file 1752-0509-7-6-S4.png]

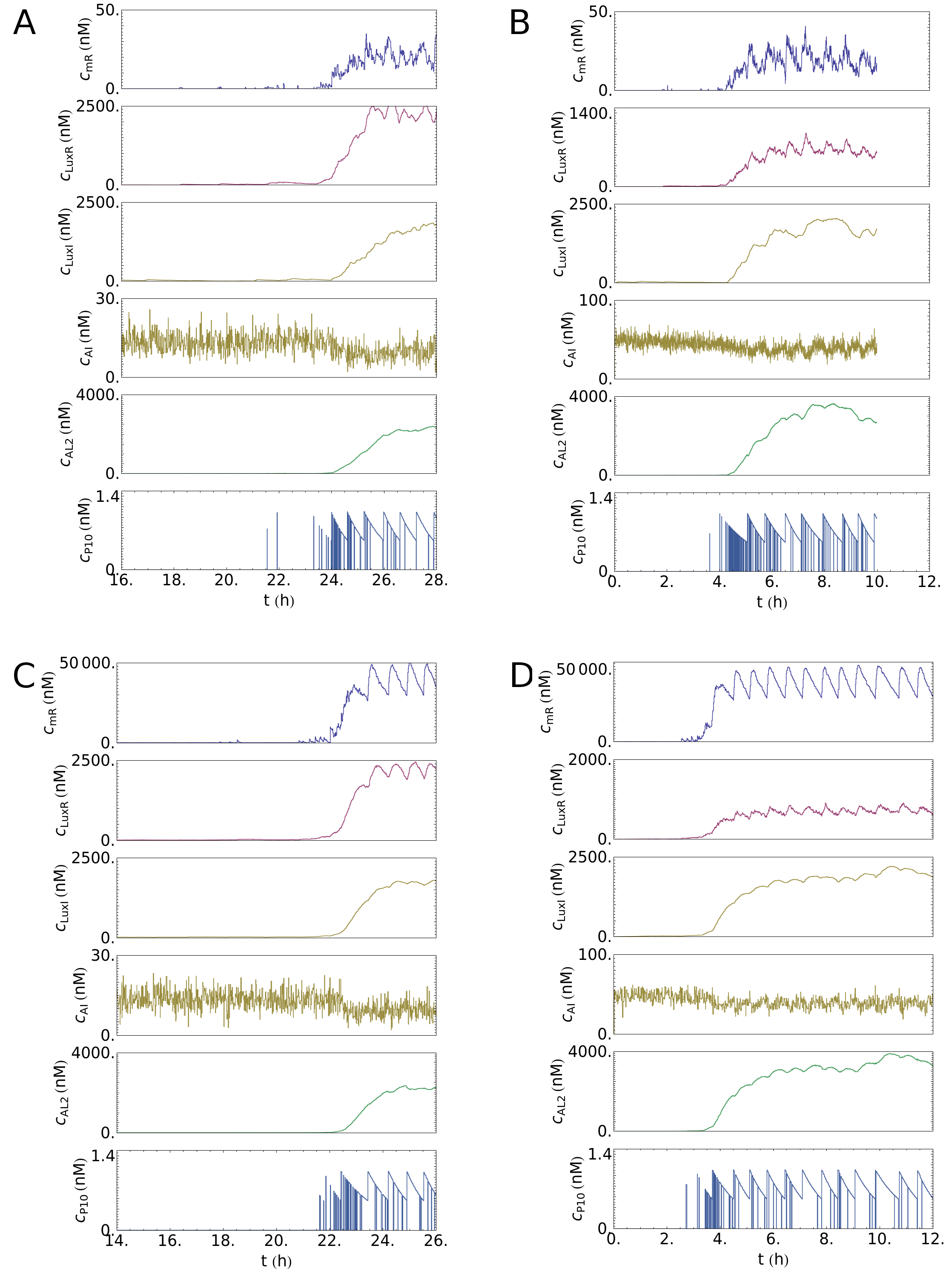

Supplement: Additional file 5 — Figure S3. Trajectory of chemical species in individual cells. Trajectory of chemical species LuxR mRNA (mR), LuxR, LuxI, intracellular autoinducer (AI), regulatory complex (LuxR·AI)2(AL2) and promoter bound to complex (P10), in an individual cell for the following control parameter and burst size values: (A) cA∗=15nM,bR=bI=20, (B) cA∗=50nM,bR=bI=20, (C) cA∗=15nM,bR=bI=0.01, (D) cA∗=50nM,bR=bI=0.01. [file 1752-0509-7-6-S5.png]
